# Comparison of the Content of Selected Bioactive Components and Antiradical Properties in Yoghurts Enriched with Chia Seeds (*Salvia hispanica* L.) and Chia Seeds Soaked in Apple Juice

**DOI:** 10.3390/antiox10121989

**Published:** 2021-12-14

**Authors:** Beata Drużyńska, Rafał Wołosiak, Monika Grzebalska, Ewa Majewska, Marta Ciecierska, Elwira Worobiej

**Affiliations:** Department of Food Technology and Assessment, Division of Food Quality Assessment, Institute of Food Sciences, Warsaw University of Life Sciences (WULS-SGGW), 159C Nowoursynowska Street, 02-776 Warsaw, Poland; beatadr2011@gmail.com

**Keywords:** chia seeds, bioactive compounds, antioxidant properties, yoghurt with chia seeds, HPLC-DAD

## Abstract

Due to the fact that consumers are looking for new, health-promoting products, there is a growing interest in various ingredients with a high biological activity that could enrich conventional foods. As is known, chia seeds are a rich source of various health-promoting compounds. The objective of this study was to determine the content of selected biologically active compounds and their antioxidant properties by means of DPPH^●^, ABTS^+●^, and the ability to chelate Fe (II) ions in chia seeds and yoghurts with the addition of these seeds and seeds soaked in apple juice. It was found that chia seeds are a rich source of bioactive ingredients with beneficial effects on human health—especially polyphenols. All the extracts showed antioxidant properties against the radicals used. The addition of seeds to yoghurt contributed to the presence of polyphenols, while soaking in apple juice resulted in a higher content of polyphenols in yoghurts. The enriched yoghurt extracts showed antioxidant properties against DPPH radicals and the ability to chelate Fe (II) ions. The addition of seeds soaked in apple juice significantly influenced the antioxidant activity against ABTS radicals. The addition of seeds (plain and soaked) did not cause significant changes in the pH of the yoghurts.

## 1. Introduction

Polyphenols are considered to be one of the most important groups of bioactive compounds in food and one of the strongest natural antioxidants. Such properties are mainly possessed by compounds that contain hydroxyl groups in their structure (polyphenols, tocopherols, ascorbic acid). Hydroxyl groups participate in the reduction of free radicals. Free radicals are atoms or molecules that have an unpaired electron on the valence orbital. This feature makes them highly reactive. Radical reactions take place very dynamically and often as chain reactions [1]. Such a mechanism leads to the destruction of biologically active molecules that are found in the environment of free radicals, e.g., proteins. This process underlies most of what we call civilization diseases: diabetes, atherosclerosis, neurodegenerative diseases (Alzheimer’s and Parkinson’s), or cancer [2,3].

Due to their structure, including aromatic acidic rings with attached -OH groups, polyphenols are characterized by very good antioxidant properties. They easily release an electron or hydrogen atoms and become free radicals themselves. The phenolic radical that results from such a reaction is a stable molecule that does not initiate oxidation reactions. The antiradical activity of polyphenols can take place in various ways: the inactivation of free radicals by donating an electron or a hydrogen atom; the reduction of other reactive oxygen species such as peroxides to harmless compounds; complexing with metals that catalyze oxidative reactions; and regenerating membrane antioxidants such as α-tocopherol [1]. Foods containing large amounts of bioactive compounds (such as phenolics) are often referred to as super foods. Chia seeds belong to this food category.

*Salvia hispanica* L. is also commonly known as chia, chia sage, or Spanish chia [4]. By reason of low resistance to frost, chia is cultivated in tropical and subtropical climates. It is also grown in greenhouses in some parts of Europe [5]. The chemical composition of chia is not constant. It can change under the influence of many factors, for example, climate or soil composition [6]. Chia seeds are appreciated for their high fatty acids content, in particular polyunsaturated fats [7,8]. Chia seeds are currently the best natural source of ɤ-linolenic acid (18:3) [9,10]. The ratio of n-6 to n-3 acids in the seed oil is 0.32:1 [7]. In addition to fat content and composition, the fiber content of the seeds (23–35%) makes it possible to include them in the group of high-fiber-content ingredients [11,12]. Chia seeds are also a rich source of protein (16–26%) [13], and they do not contain gluten proteins [14]. Apart from this, they are also a source of minerals such as calcium, phosphorous, potassium, and magnesium and vitamins such as thiamine, riboflavin, niacin, folic acid, and ascorbic acid, which helps in ailments of various adverse conditions. Their high content of phenolics has garnered an even greater interest in these seeds. Among these compounds, chlorogenic acid, caffeic acid, *p*-coumaric acid, or gallic acid, as well as quercetin and kaempferol, can be mentioned.

The use of chia seeds in the food industry is mainly related to the production of functional foods. Chia seeds are used in the production of breads, cakes, tortillas, and breakfast products. Due to the fact that *Salvia hispanica* L. seeds do not contain gluten, products with their addition can be safely consumed by people with gluten intolerance [15]. Chia seed oil can be used as a component of dietary supplements. The seeds, because of their gelling ability after hydration, are also used as stabilizers or thickening agents in products such as mayonnaise, yoghurt, sauces, or as a substitute for oil or eggs. Moreover, these gels are used in the controlled release of pharmaceuticals as well as in nanocomposites [16]. Finally, as they efficiently absorb aqueous solutions, chia seeds can also be used as “carriers” of bioactive substances. However, it is difficult to find a description of the effects of such chia seeds modification in the literature. The stability of bioactive compounds incorporated into products with chia seeds is also unknown, and is therefore one of the goals of this study.

The objective of this study was to determine the composition and content of selected biologically active compounds present in chia seeds, the possibility of enriching a food product with chia antioxidant compounds, and changing the profile of these compounds by modifying the seeds through soaking, as well as the influence of the fermentation process on the content and the activity of these compounds in a model food product.

## 2. Materials and Methods

### 2.1. Materials

The study material was *Salvia hispanica* L. seeds produced by MyVita^®^. The country of origin of the tested seeds is Paraguay. During the test, the seeds were stored in their original package in a dry and dark place. Apple juice (100%)—clear, without pulp—was purchased at a supermarket (Fortuna limited liability company).

The study material was also natural yoghurt and natural yoghurt with the addition of seeds which was prepared as follows: 17 g of granulated, skimmed-milk powdered (S.M. Gostyń) and 850 g of high-pasteurized milk (fat 3%, protein 3.5%, TS 12%, and acidity 8.0°SH) produced by OSM Wart-Milk were weighed into 6 sterile Schotts’ bottles. The contents of the bottles were well mixed and put in a water bath at 85 °C for 5 min and mixed from time to time. After this time, 7.5 mL of starter culture was added (*S. thermophilus* and *Lactobacillus delbrueckii* subsp. *bulgaricus*) to all the bottles and mixed well. This product was incubated at 45 °C for 4–5 h. After incubation, the bottles were cooled to room temperature. Dry seeds and seeds soaked in apple juice were weighed into bottles with yoghurt so that their addition accounted for 5%. All yoghurts were spilled into jars (5 jars from each bottle). Jars were stored under refrigerated conditions for a maximum of 28 days.

Before being added to yoghurt, the seeds were exposed to UV radiation for 60 s. The lamp was placed 0.5 m above the layer of chia seeds. This made it possible to achieve a radiation intensity of 1200 μW cm^−2^ at the seed level. Chia seeds were added to the yoghurt directly and also after soaking in apple juice (2 h at room temperature, under sterile conditions, in a laboratory fume hood).

### 2.2. Methods

#### 2.2.1. Extraction

##### Acetone Extract

Acetone extracts were used to perform the determinations of total polyphenols, flavonoids, tannins, and phenolics on an HPLC system, as well as the activity against DPPH^•^, ABTS^•+^ and chelating iron ions. These extracts were prepared by weighing 10 g of the tested biological material on an analytical balance into conical flasks with a volume of 300 mL and adding 100 mL of the extraction solution, which was 70% acetone solution [17]. The extracts prepared this way were shaken on a Biosan Multi-Shaker PSU 20 shaker at room temperature. Shaking was carried out for 60 min. The extracts were then filtered into conical flasks, covered with stoppers, and secured with parafilm. The extraction was performed analogously in yoghurts after prior deproteinization.

##### Preparation of an Extract for the Determination of Carotenoids

For the determination of carotenoids, 2.5 g of the testing material was transferred to a mortar, and about 15 g of anhydrous Na_2_SO_4_, previously weighed on a technical balance, was added [18]. The content of the mortar was ground until a homogeneous mass was obtained. The grated mass was then quantitatively transferred to a 100 mL ground-glass conical flask using 25 mL of hexane. The flasks were capped, wrapped with aluminum foil to minimize their exposure to light, and mixed on a Biosan Multi-Shaker PSU 20 for 48 h. The extraction was performed analogously in yoghurts after prior deproteinization.

#### 2.2.2. Characteristics of Bioactive Substances

##### Determination of the Content of Total Polyphenols

The content of total polyphenols was determined with the Folin–Ciocalteu method [19] as follows: 300 μL portions of the acetone (70%) extract were poured into test tubes, and then 4.15 mL of deionized water, 500 μL of a 20% solution of Na_2_CO_3_, and 50 μL of Folin–Ciocalteu reagent were added to the tubes. Absorbance was measured after 20 min at a wavelength of 700 nm (Shimadzu UV-1201V spectrophotometer). The content of total polyphenols was computed using the determined standard curve and was expressed in gallic acid equivalent (GAE) per 100 g of dry matter. A set of gallic acid standards was prepared by dilution with 0 mL, 1 mL, 2 mL, 3 mL, 5 mL, 10 mL, and 20 mL of gallic acid stock solution in six 100 mL volumetric flasks and filled to 100 mL with distilled water or the preparation of standard solutions of 0, 50, 100, 150, 250, 500, and 1000 mg/L gallic acid. These solutions were handled as previously described. The standard curve showing the concentration of gallic acid vs. absorbance was constructed using Microsoft Excel, and its R^2^ value was 0.9904. The calculation of the total polyphenols was performed using the following standard curve equation: y = 0.0073x + 0.0623.

##### Determination of the Content of Total Flavonoids

The total flavonoid content was determined using the spectrophotometric method described by Zhishen et al. [20]. A total of 250 μL of the extract was mixed with 1500 μL of distilled water followed by the addition of 85 μL of a 5% solution of sodium nitrite. After 5 min, 150 μL of a solution of aluminum trichloride at 10% was added and the mixture was left resting for a further 5 min, then 500 μL of 1M NaOH and 765 μL of distilled water were added, and the mixture was finally stirred. The absorbance was measured immediately at 510 nm using a Shimadzu UV-1201V spectrophotometer. The results were expressed in quercetin equivalent per 100 g of dry matter according to the analytical curve of quercetin. For the construction of the calibration curve, a quercetin stock solution of 1000 mg/L was prepared as well as a series of standard solutions of 50, 100, 200, 500, and 1000 mg/L by serial dilutions of the stock. The absorbance of standard solutions was measured and plotted against their concentration and the linear equation obtained by Excel was used for the determination of the concentration of the total flavonoids of the extracts. The R^2^ value of the obtained linear correlation was 0.9887. The calculation of the total flavonols in the extracts was carried out using the following standard curve equation: y = 0.0543x + 0.0608.

##### Determination of the Content of Tannins

The tannin determination was performed by the reaction of vanillin in HCl [21]. A total of 200 mg of chia was weighed, and 10 mL of 1% HCl solution in methanol was added. The tubes were placed in an automatic shaker (20 min) for the extraction of tannins. Then, they were centrifuged at 2865× *g* for 20 min. Aliquots of 1 mL of supernatant were added to 2.5 mL of 1% solution of vanillin in methanol and 2.5 mL of 8% solution of HCl in methanol. The absorbance reading was held in a spectrophotometer at 500 nm. The results were expressed as milligrams of catechin per 100 g of dry matter according to the analytical curve of catechin. A set of catechin standards was prepared by dilution with 0 mL, 1 mL, 2 mL, 3 mL, 5 mL, 10 mL, and 20 mL of catechin stock solution in six 50 mL volumetric flasks and filled to 50 mL with distilled water for the preparation of standard solutions of 0, 50, 100, 150, 250, 500, and 1000 mg/L catechin. These solutions were handled as previously described. The standard curve showing the concentration of catechin vs. absorbance was constructed using Microsoft Excel, and its R^2^ value was 0.9849. The calculation of the tannins in the extracts was carried out using the following standard curve equation: y = 0.0183x − 0.0254.

##### Determination of Phenolic Acids by the HPLC-DAD Method

The quantification and identification of phenolic acids in extracts were carried out in a Shimadzu HPLC system by the method of Martinez-Cruz and Paredes-López [22]. A diode array detector (Shimadzu) was used. Phenolic compounds were separated using a Supelco C18 column, 5 μm, 150 mm × 4.6 mm. The separation was achieved as follows: the mobile phase consisted of water (solvent A) and 70% aqueous acetonitrile (solvent B). All solvents were filtered through a 0.45 μm membrane prior to analysis. The system was run with the following gradient program: 0–4 min, 0–10% B; 4–8 min, 10–15% B; 8–17 min, 15–40% B; 17–35 min, 40–100% B; and 3 min, 100% B. The flow rate was kept constant at 1.0 mL/min. The injection volume for extracts and standards was 10 μL. The standards were dissolved with 70% acetone to give serial concentrations in a range of 0.0004150–0.1950 mg/mL for phenolic acids. The standard curves were prepared using the peak areas of different concentrations (mg/mL, x-axis) and were expressed by the linear least-squares regression equation. All measurements were performed in triplicate for each assay, and the results were expressed as the mean ± standard deviation.

##### Determination of Flavonoids by the HPLC-DAD Method

The quantification and identification of flavonoids in extracts were carried out in a Shimadzu HPLC system by the method of Martinez-Cruz and Paredes-López [22]. A diode array detector (Shimadzu) was used. Phenolic compounds were separated using a Supelco C18 column, 5 μm, 150 mm × 4.6 mm. The separation was achieved as follows: the mobile phase consisted of 2% acetic acid in water (solvent A) and 2% acetic acid, 30% acetonitrile, and 68% water (solvent B). All solvents were filtered through a 0.45 μm membrane prior to analysis. The system was run with the following gradient program: 0–6 min, 0–10% B; 6–10 min, 10–15% B; 10–16 min, 15–40% B; 16–30 min, 40–100% B; and 3 min, 100% B. The flow rate was kept constant at 1.0 mL/min. The injection volume for extracts and standards was 10 μL. The standards were dissolved with 70% acetone in distilled water to give serial concentrations in a range of 0.0001–0.1 mg/mL for flavonoids standards. The standard curves were prepared using the peak areas of different concentrations (mg/mL, x-axis), and were expressed by the linear least-squares regression equation. All measurements were performed in triplicate for each assay, and the results were expressed as the mean ± standard deviation.

##### Determination of the Content of Total Carotenoids 

Samples for analyses were prepared as follows: 2.0 mL portions of the hexane extract and 8.0 mL portions of hexane were collected into test tubes. The absorbance of the solutions was measured in 1 cm glass cuvettes at a wavelength of 450 nm using a Shimadzu UV-1201V spectrophotometer [23]. Results were expressed as the content of β-carotene (mg) per 100 g of dry matter. The standard curve was prepared by weighing 180 mg of K_2_Cr_2_O_7_ on an analytical balance, dissolving it in water, and transferring it to a 500 mL volumetric flask. The absorbance of such a solution corresponds to the absorbance of a β-carotene solution with a concentration of 2.08 µg/mL. Different volumes (0.5, 1.0, 2.0, 3.0, 4.0, 5.0 mL) were transferred from the stock solution to 50 mL volumetric flasks and filled up to the mark with distilled water. The standard curve showing carotene concentration as a function of absorbance was constructed in Microsoft Excel, and its R^2^ value was 0.9998. The carotenoid content was calculated using the following standard curve equation: y = 0.0157 x − 0.0359. 

##### Determination of the Content of Ascorbic Acid

Ascorbic acid content was determined by the HPLC method (isocratic). The procedure was performed according to the method of Celic et al. [24]. A total of 5 g of sample was transferred to a 50 mL volumetric flask including 10 mL of 6% (*w*/*v*) metaphosphoric acid (Sigma). The sample was then homogenized at 20,000 rpm for 15 s and centrifuged at 14,000 rpm for 10 min at 4 °C. Five milliliters of the supernatant was filtered through 0.45 μm PTFE syringe filters and placed in an amber vial. Samples were separated on a Luna C18 column (250 mm × 4.60 mm, 5 μm from Phenomenex) at room temperature. The mobile phase was 25 mM KH_2_PO_4_ with a flow rate of 1 mL/min. L-ascorbic acid was detected at 254 nm. Quantification was made by an external standard method using an L-ascorbic acid standard (Sigma). A standard curve for the determination of vitamin C was made from a standard solution of ascorbic acid using the following concentrations: 0.1, 0.3, 0.6, 0.9, 1.5, and 3.0 mg/100 mL. The equation of this curve was calculated on the basis of the area under the peaks. All points were marked in 3 replications.

Standard curve equation: y = 6.005x + 0.0495, R^2^ = 0.9980. (1)

##### Determination of Content of Ash and Sodium, Potassium, and Calcium in Chia Seeds

About 5 g of chia seeds was weighed into a previously toasted, weighed porcelain crucible on an analytical balance. The samples were then charred on an electric plate. The samples prepared in this way were placed in a muffle furnace at a temperature of 525 ± 25 °C. The tested material was kept at this temperature until the ash contained no visible carbon particles (about 16–18 h). After this time, the samples were transferred to a desiccator and, after cooling down to room temperature, weighed on an analytical balance. Total ash was calculated from the weight difference before calcination and after calcining [25].

Sodium, potassium, and calcium were determined using the emission method of flame photometry (Ciba Corning). A sample of ash (40 mg) was dissolved in 10% hydrochloric acid (10 mL) and quantitatively transferred to a 100 mL volumetric flask. In order to eliminate the influence of phosphates on the result of the calcium determination, 5 mL of 2% lanthanum nitrate was added. For the determination of potassium, the sample was diluted four times. Standard curves were based on the known concentrations of the respective salts. The result was calculated on the basis of the equations of the standard curves and dilution curves as well as the ash content in the sample [23]. The standards were prepared by weighing the appropriate amounts of the salts of the elements to be determined, and then a stock solution was prepared by mixing solutions containing the individual salts of the elements. Then, 10 mL was withdrawn from the stock solution and transferred to 100 mL volumetric flasks (replenished with distilled water). Thus, the following concentrations were obtained: 0.25, 0.5, 1.0, 2.0, 4.0, 6.0, 8.0, and 10.0 mg of a given mineral in 100 mL of solution. The following model curves were used for the calculations:

For sodium: y = 0.616x^2^ + 15.583x + 4.6891, R^2^ = 0.9999; (2)

For potassium: y = 0.4764x^2^ + 14.266x + 2.6519, R^2^ = 0.9993; (3)

For calcium: y = 0.0222x^2^ + 9.563x − 0.5412, R^2^ = 0.9988. (4)

##### pH Value

pH (yoghurts and yoghurts with chia seeds) was measured using the HI 931400 PH Meter microprocessor from HANNA Instruments [26].

#### 2.2.3. Antioxidant Properties

##### DPPH Method

The antioxidant properties of the extracts were determined by measuring their ability to inactivate stable, synthetic DPPH radicals [27]. The method consists in reducing the stable DPPH radicals by the antiradical components contained in the extract, and then measuring the decrease in the absorbance of the radical solution as a result of the reaction. Measurement of absorbance is done at a wavelength of 562 nm. The antiradical activity was estimated by means of the following equation:

(%) Inhibition = [(Abs control − Abs sample/Abs control)] × 100(5)

##### ABTS Method

The antiradical properties of extracts were also determined by examining their ability to deactivate ABTS^•+^ [28]. The principle of the method consists of the direct generation of ABTS^•+^ by the oxidation of ABTS by potassium persulphate. The addition of an antioxidant reduces ABTS^•+^ to ABTS and decreases the intensity of the absorbance. The degree of ABTS^•+^ reduction is determined spectrophotometrically at a wavelength of 734 nm. The antiradical activity was estimated by means of the following equation:

(%) Inhibition = [(Abs control − Abs sample/Abs control)] × 100(6)

##### Iron (II) Chelating Ability Method

The iron (II) chelating ability of the compounds contained in the extracts was investigated by adding iron (II) chloride and ferrozine (Sigma) to the solutions. The absorbance of the colored complex was measured 10 min after the addition of ferrozine at a wavelength of 562 nm [29]. The chelating activity was calculated from the non-chelated iron ratio taking into account the dilution and the standard curve made of different iron concentrations with the following equation: y = 0.0157x − 0.0673, R^2^ = 0.9930.

#### 2.2.4. Statistical Analysis

All tests were performed in three repetitions. Mean values and standard deviations were calculated using the Microsoft Office Excel 2007 program. For the statistical analysis, the R Commander Program was used (R i386 3.0.3). The analysis of the significance of the mean value differences was performed after one-way ANOVA with the test at α = 0.05. The analyses of the correlation between the obtained results were also calculated at α = 0.05.

## 3. Results and Discussion

Ascorbic acid content in the analyzed chia seeds reached 1.9 mg/100 g d.m. (dry matter) (Table 1). The natural yoghurt contained 0.9 mg of ascorbic acid/100 g d.m., and after 28 days of storage, this content decreased to 0.8 mg/100 g. These differences were not statistically significant, however. The vitamin C content of the yoghurt containing chia seeds was slightly higher than that of the yoghurt alone—about 1.0 mg/100 g d.m.—and also decreased after 28 days of storage (to 0.85 mg/100 g d.m.). These differences were also not statistically significant. Comparison with the content determined in chia seeds, provided in the National Nutrient Database for Standard Reference, namely 1.6 mg/100 g d.m. [30,31], showed similar values. Yoghurt enriched with seeds soaked in apple juice showed a higher initial vitamin C content than yoghurt with basic chia seeds. However, these differences were not statistically significant. After 14 and 28 days of storage, the vitamin C content of these yoghurts was found to be fairly constant. After 28 days, yoghurt enriched with chia seeds soaked in apple juice had significantly more vitamin C than yoghurts with plain chia seeds. It is known that differences in the vitamin C content of apple juice can vary considerably depending on the type of apples used to produce the juice [32], so this effect would be cultivar-dependent, and also, as reported by Grajek [33], vitamin C content may be determined by, e.g., soil composition, type of fertilization, insolation degree, and overall climatic conditions.

The content of carotenoid compounds in the analyzed chia seeds reached 2.65 mg/100 g d.m. (Table 1). In the available literature, carotenoids were also investigated in the seeds of such oil plants as soybean, rapeseed, and flaxseed, and their contents were found to range from 0.01 to 0.05 mg/100 g [34]. Hence, compared to other oil seeds, chia seeds were characterized by a high content of these compounds. However, compared to other plants, e.g., quinoa, the content of carotenoids was not high. The amount of carotenoids gained from the seeds also depends upon the method of their separation. Ixtaina et al. [5] analyzed the β-carotene content in oil produced from chia seeds by pressing or extraction with solvents. In chia seed oil extracted with a solvent, its content accounted for about 0.058 mg/100 g, whereas in the pressed oil it accounted for about 0.121 mg/100 g. Results obtained by Ixtaina et al. [5] were lower than those achieved in the seeds in our study, and this difference was probably not only due to the fact that these authors analyzed oil made of chia seeds, but also because they determined the content of β-carotene alone.

No carotenoids were found in natural yoghurt, perhaps due to the sensitivity of the method being too low. Yoghurt enriched with chia seeds had a low content of carotenoids (0.11–0.10 mg/100 g d.m.). They were stable during storage as their amount did not change. A similar trend was also observed in the case of yoghurt with chia seeds soaked in apple juice. Due to the low content of carotenoids obtained in the yoghurts, they were not taken into account in further determinations of antioxidant activity in the product.

The content of polyphenols in the analyzed chia seeds reached 209.86 mg/100 g d.m. (Table 1). According to literature data, the content of phenolic compounds in chia seeds (originating from Mexico) ranged from 88 to 92 mg/100 g [35]. In their study, Dragovic-Uzelac et al. [36] emphasized that the content of polyphenols in plants may be affected by various factors such as plant species, the conditions of cultivation, and the conditions of storage. It may, as well, be influenced by analytical conditions, considering especially the degree of material disintegration, the extractants used, and the conditions of the extraction process. Based on the determined contents of total polyphenols in chia seeds, Kim et al. [36] suggested that the use of two different methods of polyphenols extraction from chia seeds may serve for a more accurate determination of their content. The solution proposed by Kim et al. [36] was applied by Reyes-Caudillo et al. [35] in the citied studies. Another reason behind differences in the reported results might be the presence of compounds with reducing properties in the analyzed samples. These compounds may react with the reagent used for analyses and, thereby, to some extent disturb the final result of the assay [37]. Obviously, the way in which they are expressed also influences the results. In this study, the results were converted into dry matter.

The total content of polyphenols in natural yoghurts with the addition of chia seeds was observed to increase successively throughout the 28-day storage period. The differences were statistically significant. On the day of preparation, yoghurts contained about 29 mg of polyphenols/100 g d.m., while after 28 days of storage polyphenol content increased to over 36 mg/100 g d.m. Yoghurts with seeds soaked in apple juice contained significantly more polyphenols (from about 42.5 to about 48 mg/100 g of dry matter after storage) than yoghurts with plain chia seeds. This is probably due to the significant enrichment of the chia seeds with polyphenols originating from the apple juice. Apple juices are a known source of polyphenolic compounds in the literature [32,38]. Chia seed yoghurts enriched with apple juice also showed an upward trend in the amount of polyphenols during storage. The differences between the amount of polyphenols on day 0 and the amount of polyphenols on day 28 were statistically significant. The increase in polyphenol content over time may be due to the degradation of tannin complexes which were not detected after 28 days of storage in either plain seed yoghurts or soaked seed yoghurts. It is known from the literature that simpler (and therefore also decomposed) polyphenols are more easily detectable by the Folin–Ciocalteu method.

Considering the day of storage, we could discriminate only two samples when the content of polyphenols differed statistically between the analyzed yoghurts; these were samples tested at days 0 and 28, whereas an intermediate group was represented by yoghurts analyzed on day 14. Friedman and Jürgens [39] also noticed such a trend in their research.

Based on the research, it was found that the chia seeds contained 169.9 mg of flavonoids per 100 g of dry matter (Table 1). Flavonoids are the most studied group of polyphenols and account for about 60% of dietary polyphenols in plant foods with well documented antioxidant activity. After adding chia seeds, the yoghurt contained 14–16 mg of flavonoids/100 g d.m., depending on the day of storage. The trend during storage was similar to that of total polyphenols; however, the differences were not statistically significant. The addition of chia seeds soaked in apple juice to the yoghurt increased the content of total flavonoids in relation to products enriched with ordinary chia seeds (values rising during storage from 25 to 27 mg/100 g of dry matter). Significantly more flavonoids were detected in samples with modified chia seeds at all stages of storage. This trend was also confirmed in the HPLC-DAD studies.

The content of tannins in chia seeds was also determined (Table 1). It was found that the seeds contained 57.91 mg of tannins (expressed as catechin) per 100 g d.m. Depending on the method used and the cultivation conditions, the amount of these compounds may vary considerably [40]. After the yoghurt was enriched with Spanish sage seeds, the presence of tannins was also observed (14.76 mg/100 g d.m.). After 14 days of storage, their content decreased significantly, and after 28 days they were not detected. This may be related to the decomposition of tannins due to the presence of yoghurt bacteria and acidic pH [40]. The detailed mechanism of tannin breakdown is unknown and is the subject of research. Condensed tannins can be decomposed in a non-enzymatic (autoxidation) and enzymatic reaction. Various microorganisms (molds, yeasts, and bacteria) produce enzymes that enable the breakdown of tannins, some of which are released into the reaction medium. Lactic acid bacteria also belong to the microorganisms that decompose tannins [41]. The results of the HPLC analysis (Table 2) showed the presence of catechin and epicatechin, which were not found in the analysis of pure chia grain flavonoids. This phenomenon occurred in both the yoghurt containing plain chia seeds and that with chia seeds soaked in apple juice.

The extracts were analyzed by the HPLC method with a diode array detector. In the first stage, phenolic acids were determined. It was found that chia seeds contained the most gallic acid (17.72 mg/100 g d.m.) and caffeic acid (12.72 mg/100 g d.m.) (Table 2). In addition to these, ferulic, *p*-coumaric, chlorogenic, and cinnamic acids were also found. The presence of all phenolic acids found in the chia seeds was also confirmed in the yoghurts. Their content before storage ranged from 3.16 for gallic acid to 0.95 mg/100 g d.m. for *p*-coumaric acid. There were no statistically significant changes in the content of phenolic acids during storage, neither after 14 days nor after 28 days. A similar content of phenolic acids was also found in other studies [42,43].

Adding chia seeds soaked in apple juice to yoghurts increased the content of phenolic acids characteristic of apples: *p*-coumaric acid and chlorogenic acid. These differences were statistically significant. A statistically significant decrease in the content of cinnamic acid was noticed, which may be related to its small amount in apple juice. This has also been confirmed in the literature [44].

The content of some flavonoids was also determined by the HPLC method. Among the tested compounds, myricetin was found the most at 27.21 mg/100 g d.m. All the flavonoids found in chia seeds were also found in the enriched yoghurt. During the storage of the yoghurt, a statistically significant decrease in the content of apigenin, quercetin, myricetin, and kaempferol was found, while rutin remained at a similar level.

The presence of catechin and epicatechin, which was not found in chia seeds, was also detected. They were detected in yoghurts with the addition of chia seeds. As suggested above, they may have appeared due to the decomposition of condensed tannins (built of the flavan-3-ol units). This was confirmed by the results of the spectrophotometric method. It was also found that yoghurt supplemented with chia seeds was enriched relatively more in phenolic acids than in flavonoids. The flavonoids may have undergone partial biotransformation as a result of the lactic fermentation process. This is also confirmed by other works [45,46]. Lowering the pH of the environment may transform the flavonoids into derivatives that may have both weaker and stronger antiradical properties. The overall effect of these transformations can therefore be difficult to capture [47]. In this study, the transformations of these compounds did not seem to have a statistically significant influence on the antiradical properties.

The addition of chia seeds soaked in apple juice increased the content of flavonoids characteristic of apples: quercetin, catechin, and epicatechin [48]. An increase in rutin content was also observed; however, this change was not statistically significant. Additionally, the presence of phloridzin was found—a compound also characteristic for apples [47]—which was not found earlier. As a result, after 28 days of storage the total amount of flavonoids in this product was 55% higher than that of the yoghurt enriched with plain chia seeds.

In the study we also determined the content of ash and three nutritionally important elements: sodium, potassium, and calcium (Table 1). The highest amount of ash was found in chia seeds at 4.14 g/100 g d.m. Natural yoghurt had an ash content of 0.70% d.m., while in the seed-enriched yoghurt it was significantly raised to 1.32% d.m. The ash content of yoghurts with the addition of soaked chia seeds was even higher (1.92% d.m.), which was a statistically significant difference to the ash content of yoghurt with basic chia seeds.

The highest amount of sodium was found in chia seeds at 193 mg/100 g d.m. (Table 1). The sodium content therefore slightly increased in yoghurt enriched with seeds (from 34 to 40 mg/100 g d.m.); however, it was not a statistically significant change. It was found that the sodium content in yoghurt with chia seeds enriched with apple juice underwent no further statistically significant increase.

Both chia seeds and yoghurt contained high levels of potassium: 1060 and 140 mg /100 g d.m., respectively (Table 1). The enrichment of yoghurt with chia seeds significantly increased the potassium content of the product from 140 to 189 mg/100 g d.m. The addition of seeds soaked in apple juice resulted in a further significant increase in potassium content (by 74%) compared to yoghurt with plain chia seeds. It is known that apples and apple juices are a good source of this macronutrient [49].

Chia seeds are also a rich source of calcium (Table 1). It was found in seeds at the level of 360 mg/100 g d.m. Natural yoghurt was also a good source of calcium (120 mg/100 g d.m.). Adding chia seeds to yoghurt significantly increased the calcium content in the product to 137 mg/100 g d.m. The addition of seeds soaked in apple juice did not affect the calcium content in the product. It was at a similar level to that in yoghurt with plain chia seeds (135 mg/ 100 g d.m.). Similar sodium and potassium contents in chia seeds were found by Pereira da Silva et al. [45] in their research, while Prathyusha et al. [50] reported a similar calcium content.

The content of sodium and potassium is very important for the functioning of the human body. These elements take part in the regulation of the osmotic pressure of cells, which ensures the maintenance of the acid–base balance. However, their dietary content is often biased in favor of sodium, so taking into account the nutritional value it is appropriate to include foods with a high potassium to sodium ratio. Conversely, an adequate presence of calcium in the diet is a known factor in bone stability and in preventing osteoporosis [51].

Acetone extracts from chia seeds exhibited an over 95% capability to inactivate stable DPPH radicals (Table 3). Research conducted by Azeem et al. [52] demonstrated ethanolic extracts from chia seeds to be characterized by an over 85% capability to inactivate these radicals. In addition, the antioxidative activity of chia seeds extract was higher than the activity of a synthetic antioxidant (butylhydroxyanisole (BHA)). Also, Martinez-Cruz and Paredes-López [22] showed a high capability (68.8%) of chia seeds to inactivate DPPH radicals. These authors explained the good antioxidative properties of chia seeds by the high content of bioactive compounds, polyphenols in particular, but also by the presence of antioxidants soluble in both the water and the lipid phase and thus capable of inactivating radicals in both phases.

The ability to inactivate DPPH radicals determined in the extracts of natural yoghurts before storage was relatively high and reached approximately 30%, but it decreased in the successive days of storage. Contrary to this, in the case of yoghurts enriched with chia seeds, the capability to inactivate DPPH radicals was observed to increase with time. On the last day of storage, it reached about 38% and was higher by 4 percentage points compared to day 0. Hence, at the final stage of storage, the antioxidative activity of the enriched yoghurts was higher by about 14 percentage points than that of the natural yoghurts. A similar tendency was observed in the case of the addition of chia seeds soaked in apple juice. The differences in antiradical activity between plain chia seed yoghurts and soaked seed yoghurts were not statistically significant. This increase in antiradical activity may be caused by the decomposition of tannins, thereby exposing more hydroxyl groups which increase the scavenging capacity by both hydrogen transfer and single electron transfer. As in the case of polyphenols, the one-way ANOVA showed statistically significant differences (at α = 0.05) in the antioxidative activity of both the natural and enriched yoghurts against DPPH radicals during storage. Considering the effect of the day of storage, no significantly different groups were discriminated.

To determine a potential relationship between the content of determined antioxidants in chia seeds or their products and the capability to inactivate stable DPPH radicals, the respective results were subjected to correlation analysis. The statistical analysis showed that the content of polyphenols had a statistically significant (α = 0.05) effect on the antioxidative activity of chia seeds and yoghurts with their addition. A similar correlation test was conducted to find a correlation between vitamin C content in the seeds or products and their capability to inactivate DPPH^•^. Its results showed that vitamin C content also had a statistically significant (α = 0.05) effect on the antiradical activity of chia seeds.

The analyzed extracts from chia seeds exhibited a 36% capability to inactivate ABTS radical cations (Table 3). In the study conducted by Reyes-Claudillo et al. [35], the greatest amount of ABTS^+•^ was inactivated by compounds extracted from chia in the first minute of reaction. In the successive minutes, the content of ABTS^+•^ in the analyzed solution decreased insignificantly. After 12 min of reaction, about 87–96% (depending on variety) of ABTS^+●^ was inactivated. The capability to inactivate radical cations after 6 min, i.e., the period after which the ABTS assay was conducted in our study, already reached approximately 87–96%. These values are higher compared to the results achieved in our study, indicating a different antioxidant profile and a lower content of ABTS^+●^-reactive compounds in the seeds used by us for yoghurt enrichment. The antiradical activity against ABTS^+●^ was also determined for the analyzed yoghurts. The natural yoghurts showed no capability to inactivate ABTS^+●^, indicating a lack of antioxidants that could be extracted from yoghurt with aqueous acetone that were active against ABTS radical cations at the concentrations used. The minimal activity against these radicals, reaching 0.9%, was only observed in the yoghurt enriched with chia seeds.

In yoghurts with the addition of seeds soaked in apple juice, the ability to deactivate ABTS radicals was found at the level of about 10%. This activity did not change during storage. The appearance of such activity against ABTS radicals was the result of changing the antioxidant profile by the introduction of new compounds from apple juice. This may be particularly due to specific phenolics with the ability of fast reaction with ABTS^+●^ as well as the synergistic effects of the presence of various antioxidants, some of which could regenerate particularly active compounds. This role could also be played by ascorbic acid, which, despite its relatively low content, could show a supporting effect after soaking the seeds in apple juice. This can be confirmed to some extent in the results of the correlation analyses performed. The content of polyphenols was found to have a statistically significant (α = 0.05) effect on the capability of extracts to inactivate ABTS^+●^ while at the same level of significance vitamin C content had no statistically significant effect on this capability of the analyzed extracts. The reaction of flavonoids with ABTS radicals is very complex and multistage. The initial inactivation of the ABTS molecule results in a modification of the antioxidant. The modified compounds may still react with the radicals. However, it should be considered that the reaction of ABTS radicals with some flavonoids may result in products with stronger antioxidant properties than the parent compounds. The sum of the antiradical activity is then the sum of the activity of the test substance and the products of its oxidation [53].

The distinctly higher activity towards DPPH radicals than ABTS radical cations results mainly from the different molar ratio of the antioxidant to the radical molecules, which is related to the different structure of the assay. Moreover, both radicals can be deactivated by both electron transfer (SET) and hydrogen atom transfer (HAT). The latter method is especially preferred in the case of DPPH radicals [54]. It seems that the applied reaction medium (with a predominance of methanol, but also containing acetone and releasing hydrogen transfer water molecules) can effectively facilitate the deactivation of radicals by the HAT mechanism, without eliminating the possibility of using the SET mechanism in parallel.

The presence of transition metal ions (such as iron or copper ions) in the reaction environment is an important pro-oxidative factor. These metals catalyze the decomposition of low-reactive pro-oxidants (hydrogen peroxide, fatty acid hydroperoxides) to highly-reactive free radical products (mainly hydroxyl radical). This can lead to impaired cell function and, in food, to catalyzing very unfavorable autoxidation. Therefore, the binding of transition metal ions is an evolutionarily important supporting mechanism of antioxidant activity [55].

The analyzed extracts from chia seeds were able to chelate about 98% of the iron (II) present in the reaction mixture (Table 3). This ability was very high compared to studies based on other plant material; however, we found no reports in the available literature regarding the determination of the chelating ability of iron ions by chia seed extracts.

Contrary to the methods discussed above, Fe (II) chelating ability was very uniform, regardless of sample modification and storage period. In the case of natural yoghurts and yoghurts enriched with chia seeds, the capability to chelate iron (II) ions was still very high and exceeded 98%, and no statistically significant differences were observed between the chelating capability values. In addition, in the entire 28-day storage period, the Fe (II) ions chelating capability increased insignificantly in the case of natural yoghurts. In turn, in the case of yoghurts enriched with chia seeds, the chelating ability decreased minimally during the first 14 days of storage, and increased slightly on the 28th day. The ability to chelate iron (II) ions was slightly increased in yoghurts with the addition of chia seeds soaked in apple juice. The differences were not statistically significant, however. These products also showed no significant change in chelating capacity during storage. The relationship between the capability of extracts to chelate iron (II) and their content of polyphenols was checked, but no correlation was found (α = 0.05). This suggests that the ability to bind iron ions is primarily related to the presence of specific phenolic compounds, and not to the total polyphenol content, which is rather typical of commonly used methods for determining antioxidant activity, e.g., against stable radicals [56].

**Figure 1:**
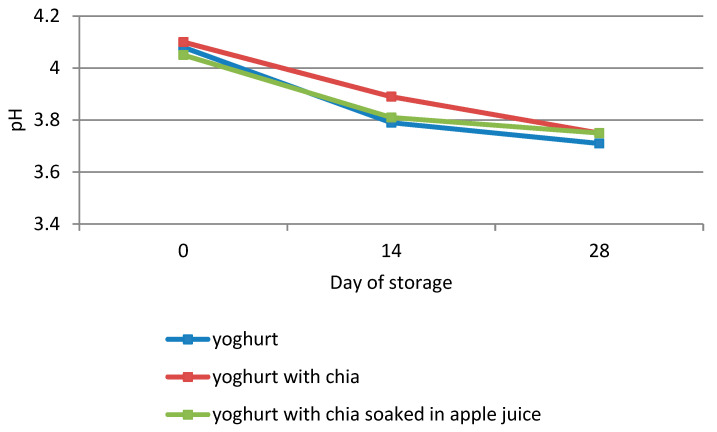
Change of yoghurt pH values during storage.

One of the parameters controlled in yoghurts during the 28-day storage was their pH. The initial pH value of natural yoghurt was 4.08 (Figure 1), and it was observed to decrease after 14 days of storage. Within the successive 14 days of storage it was still decreasing, although more slowly, and reached 3.71 on day 28. A similar tendency was observed for yoghurts with chia seeds (plain and soaked) with an initial pH of 4.05–4.10, which decreased to 3.75 on the last day of storage. Considering the above, it may be proposed that the pH value of yoghurts decreased during storage due to the activity of lactic acid bacteria, gradually slowed down over time by the use of refrigeration conditions and the accumulation of lactic acid. It was also noticed that the pH values of the analyzed types of yoghurts were very similar throughout the storage period. This allowed us to conclude that the addition of chia seeds (plain and soaked) to yoghurts does not significantly affect the activity of the lactic acid microflora.

## 4. Conclusions

Chia seeds were characterized by a high content of such bioactive compounds as polyphenols, especially flavonoids, but also tannins and some phenolic acids. All analyzed chia seed extracts showed a high ability to inactivate stable DPPH radicals and chelate pro-oxidative iron (II) ions. They also showed antiradical activity against the ABTS radical cations, but due to the assay limits it was lower than in the case of both previous methods.

The introduction of chia seeds to yoghurts resulted in their enrichment with all the groups of phenolics. The obtained results suggest, however, that these compounds behave unequally under the conditions of low pH and active microflora. Contrary to stable phenolic acids and flavonoids, the condensed tannin fraction underwent what was probably a predominantly bacterial decomposition, which resulted in a quantitative and qualitative increase in the flavonoid fraction. In yoghurts enriched with chia seeds soaked in apple juice, a higher content of total polyphenols, total flavonoids, as well as of individual phenolic acids and flavonoids characteristic of apples and apple juice was found.

Yoghurts enriched with chia seeds (plain and soaked) were able to inactivate stable DPPH radicals, and this ability was obviously lower than in the case of the seeds alone. In contrast to plain yoghurt, the activity of chia yoghurts against DPPH^●^ tended to increase during storage. Only the yoghurts with the addition of chia seeds soaked in apple juice showed significant ABTS radical cations activity. The ability of enriched yoghurts to chelate iron (II) ions was almost identical and did not change throughout the storage period. Therefore, taking into account both the content of antioxidant compounds and their activity, enriching yoghurts with chia seeds significantly increases their bioactivity, and soaking the seeds in apple juice allows it to be further developed. This is a particularly interesting feature due to the large selection of different juices containing health-promoting ingredients that can thus be incorporated into a new product. The method used ensures their stability, except for more complex polyphenols, but these are decomposed into more active flavonoids as a result of fermentation and storage.

The activity of food-derived antioxidants has limitations. It allows the inhibition of oxidative reactions unfavorable in terms of product stability and causing the loss of important nutrients and desirable sensory properties as well as the formation of toxic reaction products. It supports the organisms striving to achieve the redox equilibrium, the loss of which is associated with the development of many civilization diseases, e.g., atherosclerosis, diabetes, or neoplasms. On the other hand, an excess of antioxidant ingredients can even cause pro-oxidative effects in situ or reduce the bioavailability of some nutrients. It seems, however, that taking into account the results obtained in the study, yoghurts with the addition of chia seeds show a good level of antioxidant properties, and the susceptibility of the seeds to the introduction of other ingredients by soaking allows for their targeted modification. Such a use of chia seeds, both as a source of important ingredients and as a carrier of additional compounds, makes it possible to develop new functional products with a designed composition and activity.

## Figures and Tables

**Table 1:** The content of bioactive compounds in chia seeds, natural yoghurt, and yoghurt with the addition of chia seeds.

Type of Research Material	Total Polyphenols	Total Flavonoids	Tannins	Ascorbic Acid	Total Carotenoids	Ash	Na	K	Ca
	mg/100 g Dry Matter								
Chia seeds	209.86 (±1.04)	169.92 (±1.07)	57.91 (±3.08)	1.90 (±0.05)	2.65 (±0.24)	4140 (±0.1)	192.8 (±0.6)	1059.8 (±0.9)	358.7 (±0.6)
Natural yoghurt 0 days 14 days 28 days	* nm * nm * nm	* nm * nm * nm	* nm * nm * nm	0.91 (±0.04) ^a^ 0.85 (±0.09) ^a^ 0.81 (±1.04) ^a^	** nd ** nd ** nd	700 (±0.1) ^A^	34.5 (±0.8) ^A^	140.4 (±1.0) ^A^	120.2 (±0.6) ^A^
Yoghurt with chia 0 days 14 days 28 days	29.19 (±1.00) ^aA^ 32.81 (±1.04) ^abA^ 36.65 (±0.99) ^bA^	14.12 (±1.04) ^aA^ 15.34 (±0.94) ^aA^ 16.02 (±0.94) ^aA^	4.76 (±2.54) ^aA^ 4.34 (±1.02) ^bA^ nd	1.01 (±0.04) ^aA^ 0.92 (±0.08) ^aA^ 0.85 (±0.09) ^aA^	0.11 (±0.004) ^aA^ 0.10 (±0.002) ^aA^ 0.10 (±0.004) ^aA^	1320 (±0.2) ^B^	39.6 (±0.3) ^A^	189.2 (±0.9) ^B^	137.3 (±0.9) ^B^
Yoghurt with soaked chia 0 days 14 days 28 days	42.54 (±2.04) ^aB^ 43.42 (±1.56) ^abB^ 47.66 (±2.99) ^bB^	25.42 (±2.04) ^aB^ 25.34 (±1.17) ^aB^ 27.02 (±1.52) ^aB^	4.92 (±2.54) ^aA ^ 4.48 (±2.54) ^aA^ ** nd	1.21 (±0.09) ^aA^ 1.21 (±0.08) ^aB^ 1.07 (±0.08) ^aB^	0.10 (±0.002) ^aA^ 0.11 (±0.002) ^aA^ 0.10 (±0.003) ^aA^	1920 (±0.3) ^C^	40.8 (±2.6) ^A^	254.4 (±2.0) ^C^	135.2 (±1.9) ^B^

* nm—not marked; ** nd—not detected. Lowercase letters in the column indicate statistically significant differences within one group. The differences between the mean values marked with different letters in the column are statistically significant (*p* < 0.05). Capital letters in the column indicate statistical differences between product groups. The differences between the mean values marked with different letters in the column are statistically significant (*p* < 0.05).

**Table 2:** Composition of phenolic acids and flavonoids in chia seeds and yoghurt enriched with these seeds at different storage times (mean of three replicates, mg/100 g dry matter).

	Chia Seeds	Yoghurt with Chia Seeds			Yoghurt with Soaked Chia Seeds		
		0 days	14 days	28 days	0 days	14 days	28 days
Phenolic acids:							
Caffeic acid	12.72 (±1.17)	2.51 (±0.64) ^aA^	2.23 (±0.09) ^aA^	2.21 (±0.15) ^aA^	2.72 (±0.24) ^aA^	2.57 (±0.61) ^aA^	2.16 (±0.66) ^bA^
Ferulic acid	7.21 (±1.04)	1.27 (±0.04) ^aA^	1.02 (±0.24) ^aA^	0.87 (±0.09) ^aA^	1.22 (±0.04) ^aA^	0.98 (±0.14) ^aA^	0.65 (±0.08) ^bA^
Gallic acid	17.72 (±1.09)	3.16 (±0.09) ^aA^	2.92 (±0.09) ^aA^	2.97 (±0.13) ^aA^	3.23 (±0.71) ^aA^	2.87 (±0.45) ^aA^	2.21 (±0.23) ^bB^
*p*-coumaric acid	5.71 (±1.23)	0.95 (±0.09) ^aA^	1.05 (±0.31) ^aA^	0.93 (±0.01) ^aA^	1.54 (±0.25) ^aB^	1.49 (±0.29) ^aB^	1.03 (±0.31) ^bA^
Chlorogenic acid	7.22 (±1.02)	1.23 (±0.08) ^aA^	1.03 (±0.19) ^aA^	1.19 (±0.08) ^aA^	2.54 (±0.64) ^aB^	2.38 (±0.56) ^aB^	1.99 (±0.19) ^bB^
Cinnamic acid	9.71 (±1.17)	1.51 (±0.08) ^aB^	1.54 (±0.21) ^aB^	1.82 (±0.43) ^aB^	0.87 (±0.09) ^aA^	0.83 (±0.11) ^aA^	0.54 (±0.09) ^aA^
Flavonoids:							
Apigenin	9.71 (±1.55)	1.01 (±0.17) ^aA^	0.94 (±0.09) ^aA^	0.43 (±0.02) ^bB^	1.12 (±0.11) ^aA^	0.89 (±0.04) ^aA^	0.71 (±0.09) ^aA^
Quercetin	15.11 (±1.12)	1.66 (±0.02) ^aA^	0.52 (±0.04) ^bA^	0.48 (±1.07) ^bA^	3.56 (±0.87) ^aB^	3.23 (±0.46) ^aB^	2.45 (±0.50) ^bB^
Myricetin	27.21 (±1.176)	2.41 (±0.07) ^aA^	1.56 (±0.12) ^bA^	1.12 (±0.17) ^bA^	2.05 (±0.07) ^aA^	1.67 (±0.09) ^aA^	1.10 (±0.09) ^bA^
Kaempferol	10.71 (±1.01)	1.12 (±0.19) ^aA^	0.41 (±0.01) ^bA^	0.42 (±0.04) ^bA^	0.89 (±0.10) ^aA^	0.56 (±0.12) ^aA^	0.39 (±0.17) ^abA^
Rutin	10.21 (±1.52)	1.09 (±0.22) ^aA^	1.03 (±0.10) ^aA^	0.92 (±0.04) ^aA^	1.34 (±0.45) ^aA^	1.30 (±0.46) ^aA^	1.27 (±0.08) ^aA^
Catechin	* nd	1.11 (±0.27) ^aA^	1.29 (±0.11) ^aA^	1.10 (±0.11) ^aA^	2.76 (±0.61) ^aB^	2.65 (±0.58) ^aB^	2.23 (±0.50) ^aB^
Epicatechin	* nd	0.96 (±0.13) ^aA^	1.02 (±0.13) ^aA^	1.06 (±0.09) ^aA^	3.54 (±0.34) ^aB^	3.20 (±0.48) ^aB^	2.16 (±0.49) ^bB^
Phloridzin	* nd	* nd	* nd	* nd	2.45 (±0.41) ^a^	2.19 (±0.36) ^a^	1.89 (±0.36) ^ab^

* nd—not detected. Lowercase letters in the lines indicate statistically significant differences within one group. The differences between the mean values marked with different letters in the line are statistically significant (*p* < 0.05). Capital letters in the lines indicate statistical differences between product groups. The differences between the mean values marked with different letters in the line are statistically significant (*p* < 0.05).

**Table 3:** Results of measurement of antioxidant activity of chia seed extracts and yoghurt extracts enriched with chia seeds measured by three methods.

	DPPH (%)	ABTS (%)	Chelating Ability (%)
Chia seeds	95.6 (±2.5)	36.4 (±3.6)	98.1 (±1.9)
Natural yoghurt			
0 days	30.6 (±2.1) ^b^	* nd	98.00 (±1.4) ^a^
14 days	26.9 (±2.4) ^a^	* nd	98.22 (±1.5) ^a^
28 days	24.5 (±2.5) ^a^	* nd	98.24 (±1.5) ^a^
Yoghurt with chia			
0 days	33.6 (±1.9) ^aA^	0.6 (±0.2) ^aA^	98.19 (±1.9) ^aA^
14 days	34.5 (±1.1) ^aA^	0.6 (±0.1) ^aA^	98.10 (±1.6) ^aA^
28 days	38.1 (±1.3) ^aA^	0.9 (±0.1) ^aA^	98.32 (±2.0) ^aA^
Yoghurt with soaked chia			
0 days	35.2 (±1.5) ^aA^	10.2 (±0.9) ^aB^	98.90 (±1.4) ^aA^
14 days	37.1 (±2.0) ^aA^	9.8 (±0.8) ^aB^	98.90 (±1.4) ^aA^
28 days	37.8 (±1.5) ^aA^	9.7 (±0.7) ^aB^	99.01 (±1.2) ^aA^

* nd—not detected. Lowercase letters in the column indicate statistically significant differences within one group. The differences between the mean values marked with different letters in the column are statistically significant (*p* < 0.05). Capital letters in the column indicate statistical differences between product groups. The differences between the mean values marked with different letters in the column are statistically significant (*p* < 0.05).

## Data Availability

The data presented in this study are publicly available.
